# Letter to the Editor: “Gout on the acute medical take”

**DOI:** 10.1016/j.clinme.2026.100552

**Published:** 2026-01-20

**Authors:** Temi Lampejo, Naman Bhatt

**Affiliations:** a Faculty of Life Sciences and Medicine, King’s College London, UK; b Department of Clinical Radiology, West Hertfordshire Teaching Hospitals NHS Trust, Hertfordshire, UK

Dear Editor,

We read with great interest the review article by Abhishek *et al*^1^ who, in their comprehensive review article, highlight the key clinical, biochemical and radiological features of gout as well as discussing the principles of management. The authors also discuss key differential diagnoses of gout flare and the particular challenges in the differentiation of gout flare from septic arthritis or pseudogout. The author also discusses the importance of identifying potential risks factors for infection, synovial fluid culture and blood cultures in making this differentiation. Distinguishing between gout flare and septic arthritis is also pertinent from a management perspective, as the authors highlight that neither oral nor intra-articular glucocorticoids would be administered if septic arthritis or systemic infection is suspected.

Although a rare occurrence, we would also like raise awareness that septic arthritis and crystal arthropathy can coexist, further complicating diagnosis and management. In a recent single-centre retrospective study in the USA of adult patients with a monosodium urate (MSU) or calcium pyrophosphate (CPP) native knee crystal arthropathy, of 225 knee aspirations performed (43.6% MSU and 56.4% CPP), 5.3% (n = 12) had a superimposed septic arthritis.^2^ Immunosuppression was associated with a higher incidence of coexistent septic arthritis (41.7% versus 14.6%, *p* = 0.0271). Patients with septic arthritis had a significantly higher synovial white cell count (WCC); 83.3% with concomitant septic arthritis had a synovial WCC ≥ 50,000/mm^3^ versus 9.9% of aseptic aspirations (*p* < 0.0001). Another recent study, a 10-year retrospective observational study in New Zealand of concomitant septic and crystal arthropathy found that, of 427 patients with crystal arthropathy, 7.2% had concomitant septic arthritis.^3^ The greatest predisposing factors for the coexistent pathology were immunosuppression, rheumatoid arthritis, a previous history of gout or having metalwork in the affected joint. Synovial fluid WCC showed predictive capability for superimposed infection again in their study. Of additional note in their study was that 23.6% of patients diagnosed with septic arthritis had concomitant crystal arthropathy. Septic arthritis may also therefore occur in patients with underlying gout and checking for synovial crystals is important, particularly in those with risk factors for gout or pseudogout.

In a small Spanish case series of 25 patients with coexistent septic and crystal-induced arthritis, methicillin-sensitive *Staphylococcus aureus* (MSSA, 48%), methicillin-resistant *Staphylococcus aureus* (MRSA, 12%) and *Mycobacterium tuberculosis* (12%) were the most commonly isolated organisms from synovial fluid.^4^ The commonest risk factors/underlying conditions were diabetes mellitus (24%), chronic kidney disease (CKD, 16%) and kidney transplantation (16%). There are also several cases reports in the literature of coexistent crystal arthropathy and septic arthritis, including a 74-year-old man on immunosuppressive treatment for a vasculitis who developed gout of the knee with superimposed *Nocardia farcinica* septic arthritis, and a 42-year-old male cardiac transplant recipient who developed oligoarticular *Mycobacterium kansasii* infection along with gout.^5^^,^^6^

It is unclear whether, in some individuals, crystal arthropathy predisposes to infection within the affected joint, although a large UK general population-based study has suggested that gout is associated with an increased likelihood of septic arthritis.^7^ Clinicians should be aware that in rare instances (with immune compromise being a risk factor), coexistent pathology can occur, reinforcing the importance of microbiological investigations even if there is a clear history and/or high index of suspicion of crystal arthropathy. Additionally, when concomitant septic arthritis does occur, it may occasionally be due to atypical pathogens (such as mycobacteria), particularly in the immunocompromised.

Given the similar and overlapping clinical features of crystal arthritis and septic arthritis, all individuals with a hot, swollen (native) joint should have aspirated joint fluid sent for Gram stain, culture and polarising light microscopy (for identification of crystals) in concordance with national guidance.^8^ This is important in routine clinical practice to avoid missing patients who may have either or (rarely) coexistent pathology.

## Funding

No specific funding was received for this work.

## CRediT authorship contribution statement

**Temi Lampejo:** Writing – original draft, Conceptualization. **Naman Bhatt:** Writing – review & editing.

## Declaration of competing interest

The authors declare that they have no known competing financial interests or personal relationships that could have appeared to influence the work reported in this paper.
